# First-Principles Insights on the Formation Mechanism of Innermost Layers of Solid Electrolyte Interphases on Carbon Anodes for Lithium-Ion Batteries

**DOI:** 10.3390/nano12203654

**Published:** 2022-10-18

**Authors:** Qing Peng

**Affiliations:** 1 Physics Department, King Fahd University of Petroleum & Minerals, Dhahran 31261, Saudi Arabia; qing.peng@kfupm.edu.sa; 2 K.A. CARE Energy Research & Innovation Center at Dhahran, Dhahran 31261, Saudi Arabia; 3 Interdisciplinary Research Center for Hydrogen and Energy Storage, King Fahd University of Petroleum and Minerals, Dhahran 31261, Saudi Arabia

**Keywords:** solid electrolyte interphase, graphitic anode, initial decomposition, innermost layers of SEI, first-principles calculations

## Abstract

A solid electrolyte interphase (SEI) plays an essential role in the functionality and service life of ion batteries, where the structure and formation mechanism are still in the midst. Here, we investigate the initial decomposition and reactions of ethylene carbonate (EC) on the surface of a graphite anode using first-principles calculations. EC initially decomposes via the homolytic ring opening with the product of radical anion CH_2_CH_2_OCO_2_^−^. Bonding with Li, it forms a co-plane structure of CH_2_CH_2_OCO_2_Li, with a binding energy of 1.35 eV. The adsorption energy is −0.91 eV and −0.24 eV on the graphite zigzag edge surface and basal surface, respectively. Two CH_2_CH_2_OCO_2_Li molecules react to form a two-head structure of lithium ethylene dicarbonate (CH_2_OCO_2_Li)_2_, namely LEDC, which further forms a network preferring zigzag edge surfaces. Our results suggest that the first and innermost layers of the solid electrolyte interphase are CH_2_CH_2_OCO_2_Li sticking and networking on the zigzag edges of the surfaces of graphite anodes.

## 1. Introduction

Lithium-ion batteries have broad applications in portable electronic devices today due to their merits, such as a small volume, high operating voltage, high energy density, low self-discharge rates, long service life, and no memory effect [1,2,3,4,5,6]. The importance of lithium-ion batteries is also reflected by the 2019 Nobel Prize. A typical lithium-ion battery system consists of an anode, cathode, and non-aqueous liquid electrolyte. A solid electrolyte interphase (SEI) refers to the solid layer covering the anode and is widely believed to form due to the decomposition of electrolytes [7,8,9,10,11,12]. Significant to the function of Li-ion batteries, the SEI is electrically insulating yet sufficiently conductive to lithium ions [13]. Moreover, it prevents further decomposition of the electrolyte during the normal use of Li-ion batteries. Therefore, the SEI plays a critical role in influencing the performance, including cycle life, self-discharge, safety, faradic efficiency, and irreversible capacity, of Li-ion batteries [9,14,15,16,17,18,19,20,21].

The formation process of the SEI strongly depends on the type of anode and electrolyte materials, as well as the process of fabrication. Nowadays, many Li-ion rechargeable batteries employ carbon graphite as their anode materials and mixtures of ethylene carbonate (EC) with linear carbonates, such as dimethyl carbonate (DMC) and diethyl carbonate (DEC), as electrolytes. Consequently, the mechanisms of SEI formation between the EC-based electrolytes and carbon graphite has been extensively studied both experimentally [14,22,23,24,25,26,27] and theoretically [28,29,30,31,32,33,34,35,36,37,38,39], summarized in the reviews in Refs. [7,8,9,10,11,12,40,41]. It has been believed that only EC decomposes and contributes to the SEI formation, while the other components of linear carbonates merely improve the viscosity and conductivity of the electrolyte [28,29,30,31,32,33,34,35,36,37,38,39]. As a result, studies have mainly focused on the decomposition process of EC molecules. For example, Aurbach et al. investigated the surface chemistry of lithium electrodes in alkyl carbonate solutions using surface-sensitive Fourier-transform infrared (FTIR) spectroscopy and found that Li_2_CO_3_ and ROCO_2_Li were the major decomposition products of EC [42]. Furthermore, Aurbach et al. [24] experimentally explored reactions between EC and nucleophiles (such as lithium tert-butoxide) on lithiated graphite, and identified Li_2_CO_3_ and lithium ethylene dicarbonate (CH_2_OCO_2_Li)_2_ (namely LEDC) as the major decomposition products of EC under this condition. Based on their experimental evidence, Aurbach et al. proposed a three-step reduction mechanism (two-electron path) for EC decomposition on lithiated graphite electrodes. Later, Li and Balbuena showed this three-step reduction mechanism of EC thermodynamically feasible using ab initio density functional theory (DFT) and conventional transition state theory (CTST) computation methods [30]. A recent experiment suggested that the majority of LEDC in the SEI has been called into question [43].

**Figure 1:**
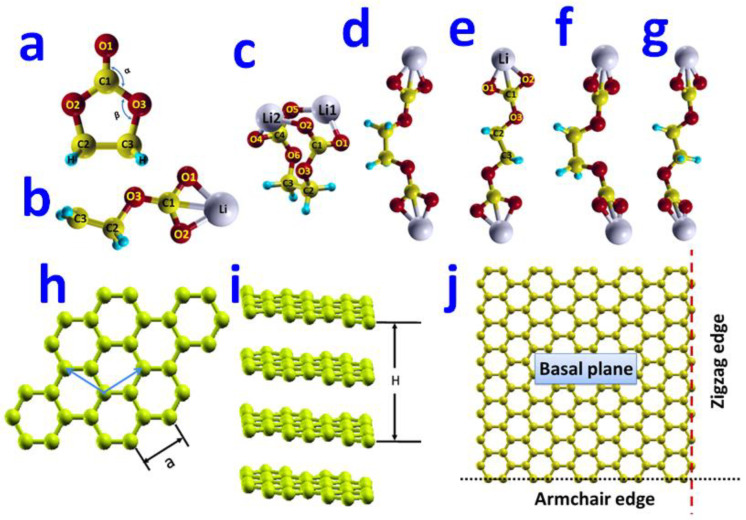
(**a**) Atomic structure of EC molecule determined by the DFT method. In the figure, red, yellow, blue balls represent oxygen, carbon, hydrogen atoms, respectively. (**b**) The most energetically favorable atomic structure of CH_2_CH_2_OCO_2_Li. In the figure, red, yellow, blue, and white balls represent oxygen, carbon, hydrogen, and lithium atoms, respectively. (**c**) *gauche*, (**d**) *cis*, (**e**) *trans*, (**f**) twisted *cis*, (**g**) twisted *trans* configuration of (CH_2_OCO_2_Li)_2_. (**h**) The two arrows form the lattice vectors for the primitive unit cell on the basal plane of graphite. (**i**) The side-view of atomic structure of the bulk graphite. (**j**) The schematic plot of basal surface, armchair edge (dotted line), and zigzag edge (dashed line).

Previous DFT studies [7,32,38] indicated that the cleavage of the O2-C2 or O3-C3 bond in Li^+^-coordinated EC is thermodynamically and kinetically more favorable than the cleavage of the O2-C1 or O3-C1 bond (Figure 1a). Therefore, the cleavage of the O2-C2 or O3-C3 bond should be the primary reductive reaction of EC in Li-ion batteries. Following this bond cleavage, radical anion CH_2_CH_2_OCO_2_^−^ can further react with Li^+^ to form lithium ethylene dicarbonate ((CH_2_OCO_2_Li)_2_) [32,39,44,45], which could constitute the main composition of the SEI [41]. It appears that this theoretical prediction is consistent with some experimental findings. For instance, Aurbach et al. [26] found that no matter what salt was used in an EC:DMC 1:1 electrolyte solution, (CH_2_OCHO_2_Li)_2_ could be found as one of the EC reduction products in the SEI, covering the graphite electrodes in EC:DMC 1:1 electrolyte solutions. In a more elaborated study, Zhuang et al. [46] compared the FTIR spectrum from synthesized (CH_2_OCHO_2_Li)_2_ and the SEI formed in 1.2 M LiPF_6_/EC:EMC electrolyte solution on a Ni electrode, concluding that (CH_2_OCHO_2_Li)_2_ was the dominant surface species on the electrodes. However, an experiment by Hardwick et al. showed that (CH_2_OCHO_2_Li)_2_ was absent in the SEI formed on seriously damaged graphite anodes and the resultant SEI was more resistive than those on normal graphite anodes [47]. Besides monomers of (CH_2_OCO_2_Li)_2_, DFT calculations [7,33] also found that it was possible for even the dimers, trimers, tetramers, and higher order *n*-polymers of lithium ethylene dicarbonate to form on the graphite anode of the lithium-ion batteries’ surfaces through intermolecular association interactions (in the form of O···Li···O). Using ab initio molecular dynamics simulations, the voltage dependence of the interfacial electrochemical process has been investigated at lithium-intercalated graphite edge planes, which concluded that electrochemical reduction exhibits potential-dependent kinetics [48]. Using the DFT and the implicit solvation theory, the lithium insertion and desorption reaction at the SEI has been studied with insights on the charge transfers [49]. A classic molecular dynamics study suggested that, during the initial dissolution step, the Li cation prefers to shed DMC molecules as opposed to losing the EC [50].

To gain knowledge about the molecular structure of the SEI, in this work we focused on studying the adsorption configuration and strength of major EC decomposition products on the surface of carbon graphite. The paper is organized as following: Section 2 describes our computational method; Section 3 presents our computation results and discussions; and Section 4 gives the conclusions.

## 2. Computation Methods

We used the density functional theory (DFT) method to determine the optimal structures of EC, CH_2_CH_2_OCO_2_Li, and (CH_2_OCO_2_Li)_2_, and the adsorption of these molecules onto the basal and edge surfaces of graphite. The DFT calculations were performed using the Vienna Ab initio Simulation Package (VASP) [51,52] code which is based on the Kohn–Sham density functional theory (KS-DFT) [53] with the generalized gradient approximations, as parameterized by Perdew and Wang, for the exchange–correlation functions [54]. The electrons explicitly included in the calculations were the (2*s*^2^2*p*^2^) electrons of carbon, (2*s*^1^) electron of lithium, (1*s*^1^) electron of hydrogen, and (2*s*^2^2*p*^4^) of oxygen. The core electrons (1*s*^2^) of carbon, lithium, and oxygen were replaced by the projector augmented wave (PAW) and pseudopotentials [55]. The cutoff energy of the plane-waves was 400 eV. The convergence criteria of the electronic self-consistent iteration were 0.0001 eV. The atomic forces were relaxed to be smaller than 0.01 eV/Å. We used a 3 × 3 × 1 Monkhorst-Pack *k*-mesh for Brillouin-zone integration for molecular adsorption on graphite surfaces. The size effect and *k*-mesh convergence were examined as summarized in Supplemental Table S1 in the Supplementary Information.

## 3. Results

### 3.1. Isolated Molecules and Pristine Surfaces

#### 3.1.1. EC Molecules

In the optimized configuration of the isolated EC molecule (shown in Figure 1a), three carbonate atoms and three oxygen atoms lay in the same plane. For this optimized EC molecule, our DFT calculations predicted the bond lengths of R_C1-O1_ = 1.21 Å, R_C1-O2_ = 1.38 Å, R_C2-O2_ = 1.44 Å, R_C2-C3_ = 1.54 Å, and the bond angles of ∠O1C1C2 = 125°, ∠C1O2C2 = 110°. (C1, C2, O1, and O2 refer to the atoms as marked in Figure 1). Our computational results agree well with the previous DFT prediction of R_C2-C3_ = 1.53 Å [56], R_C2-O2_ = 1.43 Å [30], and the experimental data of R_C2-C3_ = 1.522 Å and R_C2-O2_ = 1.457 Å [57]. 

#### 3.1.2. CH_2_CH_2_OCO_2_Li Molecule

The first step in the decomposition process of EC is to break the ring structure. This decomposition is energetically favorable [7] and leads to the formation of a radical anion. CH_2_CH_2_OCO_2_^−^. In lithium-ion batteries, a single radical anion.CH_2_CH_2_OCO_2_^−^ can further react with a Li^+^ to form the CH_2_CH_2_OCO_2_Li molecule. The configuration of the CH_2_CH_2_OCO_2_Li molecule is determined by the minimization of the total energy of the molecule. The seven examined atomistic structures of CH_2_CH_2_OCO_2_Li are displayed in Figure S1 in the Supplementary Information. Our DFT calculations found that the lowest-energy configuration of the CH_2_CH_2_OCO_2_Li molecule would assume a structure as shown in Figure 1b, in which all the C, O, and Li atoms lie in the same plane. In this optimal structure of the CH_2_CH_2_OCO_2_Li molecule, Li^+^ strongly binds to the two O atoms and one C atom with bond lengths of R_Li-O1_ = 1.85 Å, R_Li-O2_ = 1.86 Å, and R_Li-C1_ = 2.08 Å. Our results are consistent with the previous computational values of R_Li-O1_ = 1.848 Å and R_Li-O2_ = 1.864 Å [38].

For the EC decomposition reaction $\mathrm{Li}+\mathrm{EC}\to {\mathrm{CH}}_{2}{\mathrm{CH}}_{2}{\mathrm{CO}}_{3}\mathrm{Li}$, we estimated its reaction energy ΔE to be −1.35 eV. The reaction energy was calculated as the difference in potential energies between the product (CH_2_CH_2_OCO_2_Li) and the reactants (isolated EC molecule and single Li atom) and as $E^{react}=E\left(products\right)-E\left(reactants\right)$. A negative reaction energy here indicates that this reaction was exothermic.

#### 3.1.3. (CH_2_OCO_2_Li)_2_ Molecule

It is also believed that, in Li-ion batteries with two radical anions, CH_2_CH_2_OCO_2_^−^ could react with two Li^+^ to form a (CH_2_OCO_2_Li)_2_ molecule by the chemical reaction [58].

$$2\mathrm{Li}+2\;\mathrm{EC}\to 2{\mathrm{CH}}_{2}{\mathrm{CH}}_{2}{\mathrm{OCO}}_{2}\mathrm{Li}\;\to {\left({\mathrm{CH}}_{2}{\mathrm{OCO}}_{2}\mathrm{Li}\right)}_{2}+{\;C}_{2}H_{4}$$

In order to find the lowest-energy molecular structure, we have performed structure optimization calculations for the five high-symmetry configurations of the (CH_2_OCO_2_Li)_2_ molecule using the DFT method. In this work, we found that the *gauche* configuration (shown in Figure 1c) of the (CH_2_OCO_2_Li)_2_ molecule had the lowest potential energy. 

In the optimized gauche configuration, the two CH_2_OCO_2_ side chains face each other when viewing along the central C2-C3 bond. Moreover, each Li atom binds with two oxygen atoms that belong to the two different CH_2_OCO_2_ side chains. The other four low-energy configurations of the (CH_2_OCO_2_Li)_2_ molecule are listed in Figure 1d–g. Figure 1d shows a *cis* configuration, in which the two CH_2_OCO_2_Li side chains form a dihedral angle of 60° around the central C2-C3 bond. The potential energy of this *cis* configuration was found to be 1.11 eV higher than that of the *gauche* configuration. Figure 1e shows a *trans* configuration, in which the two CH_2_OCO_2_Li clusters form a dihedral angle of 180° around the central C-C bond. For this configuration of the (CH_2_OCO_2_Li)_2_ molecule, we calculated the bond lengths to be R_Li-O1_ = 1.85 Å, R_Li-O2_ = 1.85 Å, R_Li-C1_ = 2.08 Å, R_C1-O3_ = 1.36 Å, and R_C2-O3_ = 1.52 Å. Our computational results agree well with the previous DFT predictions of R_Li1-O1_ = 1.856 Å, R_Li1-O2_ = 1.868 Å, R_Li1-C1_ = 2.095 Å, and R_C1-O3_ = 1.346 Å [33]. The potential energy of this *trans* configuration is 1.12 eV higher than that of the *gauche* configuration.

The (CH_2_OCO_2_Li)_2_ molecule can form another *cis* configuration, which is distinct from the one in Figure 1d in that the two CH_2_OCO_2_Li clusters are twisted around the C_sp2_-O_sp3_ bond by 90° as shown in Figure 1f. The potential energy of this configuration was found to be 1.12 eV higher than that of the *gauche* configuration. Figure 1g shows a twisted *trans* configuration, in which the two CH_2_OCO_2_Li clusters are twisted around the C_sp2_-O_sp3_ bond by 90°, and whose potential energy was 1.14 eV higher than that of the *gauche* configuration. Thus, our result of the transition energy from *trans* (Figure 1e) to *gauche* (Figure 1c) was 1.12 eV, which agrees well with the theoretical value of 1.048 eV [45]. 

For the EC decomposition reaction, $2\mathrm{Li}+2\;\mathrm{EC}\to 2{\mathrm{CH}}_{2}{\mathrm{CH}}_{2}{\mathrm{CO}}_{3}\mathrm{Li}\;\to {\left({\mathrm{CH}}_{2}{\mathrm{OCO}}_{2}\mathrm{Li}\right)}_{2}+{\;C}_{2}H_{4}$, we estimated its reaction energy ΔE to be −7.69 eV. The reaction energy was calculated as the difference in potential energies between the products (the *gauche* configuration of (CH_2_OCO_2_Li)_2_ and the isolated C_2_H_4_ molecule) and the reactants (the isolated EC molecule and a single Li atom). The detailed calculations are elaborated in the Supplementary Information. Hence, our calculation results imply that, after decomposing into CH_2_CH_2_OCO_2_Li, electrolyte EC can further decompose to form (CH_2_OCO_2_Li)_2_ and release 4.99 eV energy in Li-ion batteries. 

#### 3.1.4. Graphite Surfaces

Graphite has a layered structure where the carbon atoms in each layer are bonded in hexagonal arrays, and the isolated suspended single layer is named graphene [59,60,61,62,63,64,65,66,67,68]. In this work, we modeled graphite using a multi-layer graphene with a stacking order of AB (hexagonal graphite or α-phase). Shown in Figure 1h, the atomic structure of such bulk graphite was determined by two parameters: *a* (the edge length of the primary unit cell (rhombohedra cell) on basal plane) and H (the height perpendicular to basal plane, Figure 1i). From our DFT calculations, we predicted *a* to be 2.464 Å and H to be 7.197 Å in our modeled bulk graphite. Compared with the experimental measurement data (*a* = 2.461 Å and H = 6.710 Å) [69], our theoretical calculations overestimated the value of *a* by 0.1 % and the value of H by 7.3%.

Figure 1h–i show that the graphite with a layer structure has two types of surfaces: basal plane surfaces and edge surfaces. The edge surfaces of graphite could assume armchair or zigzag forms [70,71,72]. It is known that the basal plane and edge surfaces of graphite could exhibit different electrochemical behaviors [73]. Hence, we modeled both types of graphite surfaces in this work. The basal surface of graphite was modeled using a super cell containing a single graphene layer (54 carbon atoms) and a vacuum region in the direction normal to the surface. In our DFT calculations of isolated and molecule-adsorbed basal plane surfaces, we used a 4 × 4 × 1 Monkhorst-Pack *k*-mesh for Brillouin-zone integration. The armchair edge surface was modeled using a super cell containing four graphene half-layers (24 carbon atoms at each layer) and a vacuum region in the direction normal to the surface. The armchair edge of graphite is marked with a black dotted line in Figure 1j. The zigzag edge surface was modeled using a super cell containing four graphene half-layers (16 carbon atoms at each layer) and a vacuum region in the direction normal to the surface. The zigzag edge of graphite is marked with a red dashed line in Figure 1j. In our DFT calculations of isolated and molecule-adsorbed armchair and zigzag edge surfaces, we used a 3 × 3 × 1 Monkhorst-Pack *k*-mesh for Brillouin-zone integration.

### 3.2. Molecule Adsorption on Graphite Surfaces

The adsorption energy $E_{ads}$ is the energy difference between the adsorption system (the complex consisting of multiple components) and the isolated configurations of the individual components. Taking (CH_2_OCO_2_Li)_2_ adsorbing on the graphite surface as an example, the adsorption energy is calculated as $E_{ads}=E({\left({\mathrm{CH}}_{2}{\mathrm{OCO}}_{2}\mathrm{Li}\right)}_{2}+graphite)-E\left({\left({\mathrm{CH}}_{2}{\mathrm{CO}}_{2}\mathrm{Li}\right)}_{2}\right)-E\left(Graphite\right)$. The adsorption energy $E_{ads}$ indicates the adhesion strength of the adsorbate molecule on the surface. A negative adsorption energy implies that the adsorbate molecule would be energetically favorable to be adducted to the surface, while a positive adsorption energy means that the adsorbate molecule would not energetically prefer contact with the surface. Consequently, a necessary condition to form a stable SEI protective layer in Li-ion batteries is the negative adsorption energy of EC decomposition molecules on the graphite surface. The more negative these adsorption energies are, the stronger the binding strength between the SEI and the graphite anodes will be. 

In order to determine the adsorption energies of the EC decomposition molecules on the graphite surfaces, we used DFT computations to predict the optimized structures and energies of the CH_2_CH_2_OCO_2_Li and (CH_2_OCO_2_Li)_2_ molecules adsorbed on the basal and edge surfaces of graphite. We further compared the attained energies of the adsorption configuration with the energies of the isolated molecules and surfaces. The adsorption structures of EC on graphite surfaces are displayed in Supplementary Figure S2 in the Supplementary Information. The adsorption of CH_2_CH_2_OCO_2_Li and (CH_2_OCO_2_Li)_2_ molecules on graphite surfaces is elaborated as follows. 

#### 3.2.1. Adsorption of CH_2_CH_2_OCO_2_Li Molecule on Graphite Surfaces

To evaluate the adhesion strength between the CH_2_CH_2_OCO_2_Li molecule and the graphite surfaces, we placed the lowest-energy configuration of the CH_2_CH_2_OCO_2_Li molecule on various locations of the graphite surface and optimized the structures using DFT calculations. In our modeled graphite surfaces, we included a 20 Å thick vacuum region in their normal direction. 

##### Basal Plane Surface

**Figure 2:**
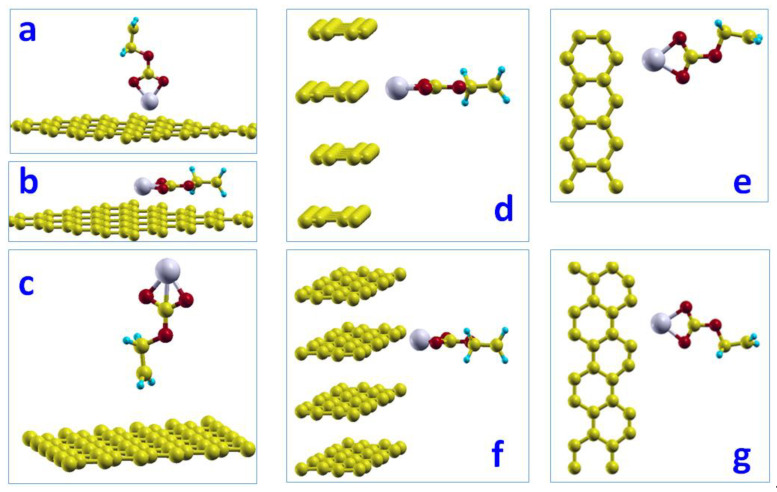
Adsorptions of CH_2_CH_2_OCO_2_Li on the graphite surface with five orientations, ordered by their adsorption energy from lowest (**a**) to highest (**c**). Configuration (**a**) has the lowest total energy and strongest adsorption with adsorption energy of −0.24 eV on basal surface. Configuration (**b**) with the C1-O2-O3 plane parallel to the basal plane surface. Adsorptions of CH_2_CH_2_OCO_2_Li on the graphite edge surfaces (**d**) zigzag top-view (**e**) zigzag side-view and (**f**) armchair top-view (**g**) armchair side-view.

We examined three distinct configurations of the CH_2_CH_2_OCO_2_Li molecule adsorbed on the basal plane surface of graphite. In the lowest-energy configuration (Figure 2a), the Li atom in the CH_2_CH_2_OCO_2_Li molecule lies 2.11 Å right above the hollow site; thus, it directly interacts with the carbon-ring atoms forming the hollow site. The distance between the Li atom and the carbon-ring atoms is 2.58 Å. The corresponding adsorption energy was determined to be −0.24 eV. Figure 2b shows the configuration that the CH_2_CH_2_OCO_2_Li molecule lies parallel to the basal plane and the Li atom lies above the hollow site. The adsorption energy associated with this configuration is −0.04 eV. Figure 2c shows the configuration that the CH_2_CH_2_OCO_2_Li molecule is flipped upside-down from Figure 2a, with an adsorption energy of 0.01 eV. Because the configuration of Figure 2a gives the lowest adsorption energy, it would be the most possible configuration for the CH_2_CH_2_OCO_2_Li molecule on the basal plane surface.

##### Edge Surfaces

We studied the adsorption of the CH_2_CH_2_OCO_2_Li molecule on the two kinds of edge surfaces of graphite, (a) zigzag edge surface and (b) armchair edge surface, as shown in Figure 2d–g. Their corresponding side-views are also shown on the side for a clear view. We found very strong adsorptions of CH_2_CH_2_OCO_2_Li on the zigzag edge surface of graphite, of −0.91 eV. The adsorption on the armchair edge surface was −0.54 eV. In this orientation, the C1-O2-O3 planes were parallel to the graphite layers, which left the channel for the migration of Li atoms and intercalation, while blocking other solvent molecules.

#### 3.2.2. Adsorption of (CH_2_OCO_2_Li)_2_ Molecule on Graphite Surfaces

Minimizing the effect of periodic images, we set the thickness of the vacuum region to 30 Å in the super cells of our modeled graphite surfaces (both basal and edge surfaces) for the adsorption of (CH_2_OCO_2_Li)_2_. Although five configurations of (CH_2_OCO_2_Li)_2_ were studied in the previous section, only the two typical configurations, *gauche* and *trans*, were subjected to the study of the adsorption on the basal plane surface, and only the *trans* configuration was selected to study the adsorption on edge surfaces for simplicity and representativeness.

##### Basal Plane Surface

**Figure 3:**
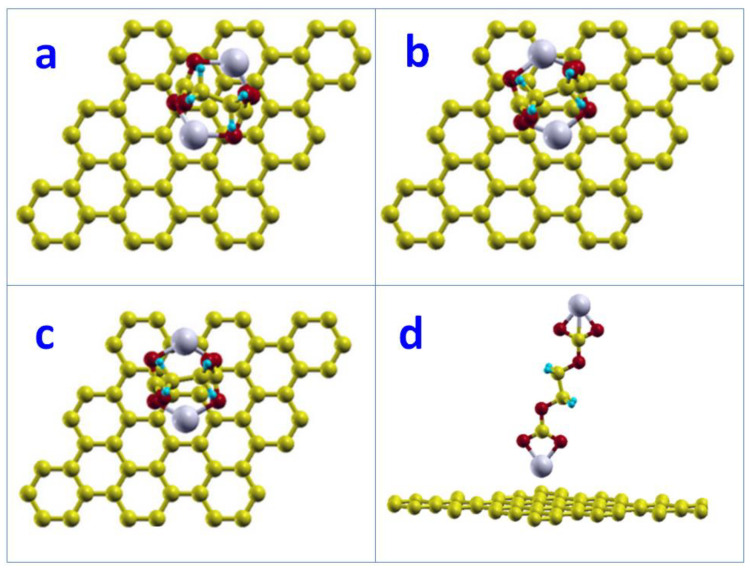
Adsorption of (CH_2_OCO_2_Li)_2_ on graphite basal plane surface: (**a**) *gauche* where two lithium atoms are on Hollow-Hollow sites. (**b**) *gauche* where two lithium atoms are on Hollow-Top sites. (**c**) *gauche* where two lithium atoms are on Hollow-Bridge sites. (**d**) *trans* configuration.

We studied the adsorption of two configurations (*gauche* and *trans*) of (CH_2_OCO_2_Li)_2_ on the graphite basal surface. When one lithium atom of *gauche* configuration sits above the hollow site of a carbon hexagon ring (center), there are three possible configurations where the other lithium atom is most likely to stand. These configurations are: (1) hollow site (H), which is on the center of another carbon hexagon ring; (2) top site (T), which is on top of a carbon atom; and (3) bridge site (B) which is the middle of the two nearest carbon neighbors. Figure 3a shows the initial *gauche* configuration on the basal plane surface where two lithium atoms are in the Hollow-Hollow configuration of the carbon hexagon ring. Figure 3b,c shows the Hollow-Top and Hollow-Bridge configurations, respectively. Figure 3d shows the *trans* configuration on the basal surface. We found adsorptions of −0.03, −0.04, −0.04, and −0.17 eV for configurations Figure 3a–d, respectively. The minimum distance between lithium and carbon atoms on the basal plane was 4.08, 4.05, 4.07, and 2.68 Å, respectively. This result indicates that the adsorption of (CH_2_OCO_2_Li)_2_ on the graphite basal surface is weak. Compared to *gauche*, the *trans* configuration has the stronger adsorption on the basal plane surface.

##### Edge Surface

**Figure 4:**
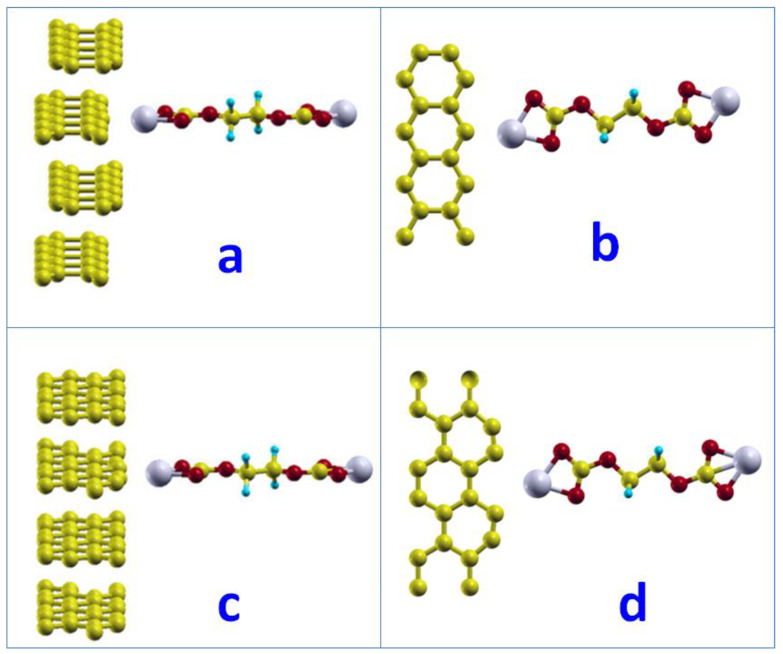
Adsorptions of (CH_2_OCO_2_Li)_2_ on the graphite edge surfaces: (**a**) zigzag surface side view; (**b**) zigzag surface top view; (**c**) armchair surface side view; (**d**) armchair surface top view.

The adsorption of (CH_2_OCO_2_Li)_2_ on the edge surface of graphite was studied on two kinds of edges, zigzag and armchair, with the *trans* configuration. Previous test examinations suggest that the parallel orientation, where the C1-O1-O2 plane of (CH_2_OCO_2_Li)_2_ is parallel to graphite basal plane, is energetically favorable. As a result, the adsorption calculations of (CH_2_OCO2Li)_2_ on zigzag and armchair edge surfaces were only carried on the parallel orientation of the *trans* configuration. Figure 4a,b are the setup of the adsorption calculations for the zigzag and armchair edge surfaces, respectively. We found that the adsorption energy was −0.49 eV on the zigzag edge surface, which was larger than the armchair edge surface, which was −0.32 eV. This result suggests that the adsorption of (CH_2_OCO_2_Li)_2_ on the zigzag edge surface is the most favorable adsorption between the basal surface, zigzag edge surface, and armchair edge surface.

### 3.3. Networking Adsorption on Graphite Surfaces

We studied several possible networks of (CH_2_OCO_2_Li)_2_ on the graphite surface. Such a network of (CH_2_OCO_2_Li)_2_ may form the backbone of the SEI layer. Therefore, the study of the formation of such a network and their adsorption on graphite surfaces is of great importance.

#### 3.3.1. Network on Basal Surface

We built up the model with one (CH_2_OCO_2_Li)_2_ linear molecule sitting 3.0 Å above the hollow site of the hexagon. Two graphene layers and a 30 Å vacuum region were used to present the basal surface on graphite. The nearest neighbor (CH_2_OCO_2_Li)_2_ molecule was 4.27 Å away.

**Figure 5:**
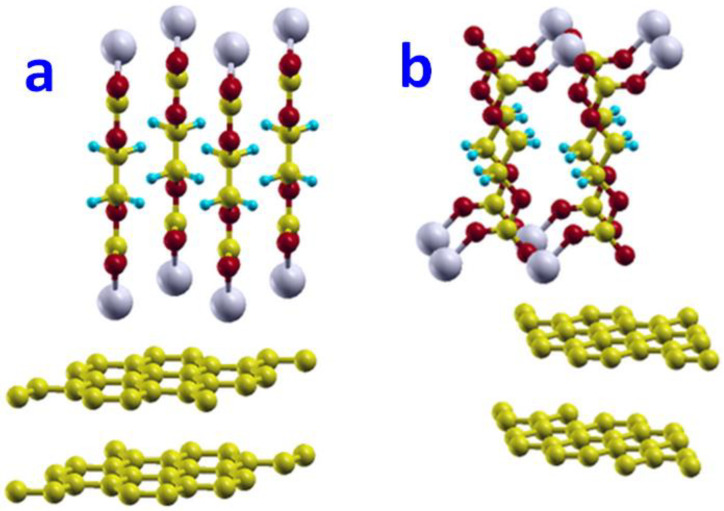
The network of (CH_2_OCO_2_Li)_2_ molecules on basal surface of graphite: (**a**) initial structure (**b**) final structure.

After full relaxation, we found that the Li atoms moved to the middle point between the (CH_2_OCO_2_Li)_2_ molecules and a network was formed. The Li atoms bonded with oxygen atoms from other (CH_2_OCO_2_Li)_2_ molecules and formed the network as shown in Figure 5. The Li-O bond was found to be 2.16 Å. The minimum distance between the lithium and carbon atoms in graphite was 3.88 Å. The adsorption energy of such networked (CH_2_OCO_2_Li)_2_ was −0.01 eV with a reaction energy of −1.39 eV. It is worth noting that the reaction energy is the energy difference between the product and the reactants $E^{react}$ = *E*(*products*) − *E*(*reactants*). For the adsorption systems, the reaction energy includes the adsorption energy (see the calculation examples in Supplementary Information).

#### 3.3.2. Network on Zigzag Edge Surface

The networking of (CH_2_OCO_2_Li)_2_ was studied on the zigzag edge surface. The surface in this model was selected to have an area of 7.4 Å × 7.4 Å. There were two layers of carbon atoms in one unit cell and 25 carbon atoms on each layer. There were 10 Å thick vacuum regions to present the surface. We used a 4 × 4 × 1 Monkhorst-Pack *k*-mesh for Brillouin-zone integration.

To reduce numerical errors during geometry optimization caused by the unphysical placement of the (CH_2_OCO_2_Li)_2_ molecules, we initially displaced the (CH_2_OCO_2_Li)_2_ molecules far away from the zigzag edge surface, about 4.0 Å, which is larger than any possible bond length among these elements between Li, O, C, and H. It turns out this is a trick to obtain a physical and reasonable optimized geometry. It is critical to set an initial large distance between (CH_2_OCO_2_Li)_2_ molecules and the zigzag surface, about 4.0 Å.

After full relaxation, the (CH_2_OCO_2_Li)_2_ molecules formed a network on the zigzag edge surface of graphite, as shown in Figure 4. The Li and a carbon atom on graphite formed a bond with a bond length of 2.14 Å, which was also the distance between (CH_2_OCO_2_Li)_2_ and the graphite zigzag edge surface. The Li-O bond was 1.95 Å. The network formed in one row along layers (perpendicular to the layer). The adsorption energy of such networked (CH_2_OCO_2_Li)_2_ on the graphite zigzag edge surface was −1.08 eV with a reaction energy of −1.66 eV. 

#### 3.3.3. Network on Armchair Edge Surface

**Figure 6:**
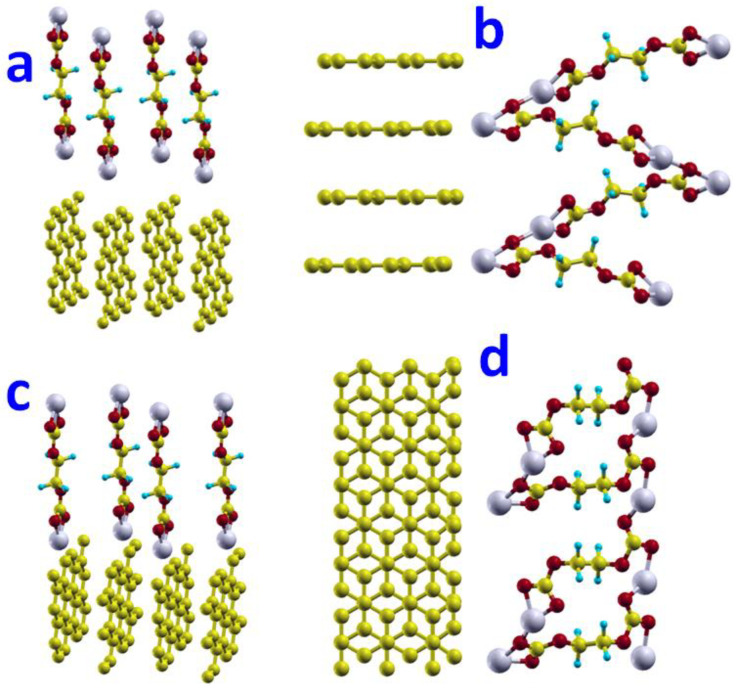
The network of (CH_2_OCO_2_Li)_2_ on the zigzag (**a**,**b**) and armchair (**c**,**d**) edge surface. Initial structure (**a**) and final structure (**b**) of network of (CH_2_OCO_2_Li)_2_ on zigzag edge surface. Initial structure (**c**) and final structure (**d**) of network of (CH_2_OCO_2_Li)_2_ on armchair edge surface.

Similarly, the networking of (CH_2_OCO_2_Li)_2_ was studied on the armchair edge surface. After full relaxation, the (CH_2_OCO_2_Li)_2_ molecules formed a network on the armchair edge surface of graphite, as shown in Figure 6c,d.

The minimum distance between the Li atom and a carbon atom on graphite is 2.45 Å, which was also the distance between (CH_2_OCO_2_Li)_2_ to the graphite armchair edge surface. The Li-O bond close to the surface was 1.9 Å, but 2.0 Å on another end. The network formed in 2D, with both rows and columns on the surfaces. The adsorption energy of such networked (CH_2_OCO_2_Li)_2_ on the graphite armchair edge surface was −0.32 eV, with a reaction energy of −1.89 eV. 

## 4. Discussions

Compared with the basal plane surfaces, the adsorptions of CH_2_CH_2_OCO_2_Li and (CH_2_OCO_2_Li)_2_ on the edge surfaces were much stronger. The reason for such a strong adsorption may lay in the presence of the dangling bonds of carbon atoms on the edge surfaces. For the same reason, the zigzag edge surfaces have a stronger adsorption to (CH_2_OCO_2_Li)_2_ than the armchair edge surfaces because the dangling bond density is higher. The ratio of dangling bonds per unit length in one layer of the zigzag edge surface to that of the armchair surface is $\sqrt{3}:1$. The precise quantity relationship between the dangling bond density and the adsorption energy needs further study and analysis. Despite this, we still can qualitatively conclude that (CH_2_OCO_2_Li)_2_, a main product of the initial electric chemical reaction, is energetically favorable to be adsorbed on the edge surface of graphite. Such an adsorption may form the first layer of the SEI on the edge surface and provide the base of other layers’ growth in further SEI forming. Compared with the isolated EC and Li molecules, the total energy gained during the binding and adsorption was 1.58 eV in this reaction path. 

To state clearly, the results of the adsorption energies of CH_2_CH_2_OCO_2_Li and (CH_2_OCO_2_Li)_2_ on the graphite basal, zigzag edge, armchair edge surfaces are listed in Table 1. The strong adsorptions on edge surfaces are because of the dangling bonds of carbon atoms on the surface. The large adsorption energy of −0.91 eV on the zigzag edge surface indicates the strong bonding between CH_2_CH_2_OCO_2_Li and the graphite surface, making this layer formed by CH_2_CH_2_OCO_2_Li electronically insulating. This layer of CH_2_CH_2_OCO_2_Li makes the innermost layer of the SEI and becomes the base for the growth of further SEI layers. This strong adsorption suggests that the SEI film is favorably formed on the edge surface. Li acts as the root and medium for the initial decomposition of EC and adsorption onto the graphite surface, forming the first and innermost layer of the SEI.

Based on our DFT calculations of the adsorption strength of EC, (CH_2_OCO_2_Li)_2_, CH_2_CH_2_OCO_2_Li on the graphite basal surface and the zigzag and armchair edge surfaces, we can propose a mechanism for the formation of the SEI on carbon anodes in E-based electrolytes. An EC molecule initially decomposes via a hemolytic ring opening through an O2C2 bond cleavage, the product of which being a radical anion, CH_2_CH_2_OCO_2_^−^, which further reacts with Li to form a stable structure of CH_2_CH_2_OCO_2_Li. In turn, it can be absorbed onto the graphite surface, with −0.24 eV on the basal surface, −0.54 eV on the armchair edge surface, and −0.91 eV on the zigzag edge surface. The absorbed CH_2_CH_2_OCO_2_Li molecule forms the first and innermost layer of the SEI. The orientation with the molecule plane (all atoms O3-C2-O3-C1-O1-O2 are coplanar), parallel to the graphite layer (see Figure 4d), ensures the function of SEI film, conducting to Li/Li+, but preventing any co-insertion of solution species.

CH_2_CH_2_OCO_2_Li is composed of the same elements and bonds with lithium ethylene dicarbonate (CH_2_OCO2Li)_2_, a well-known SEI component [13,46]. As a result, CH_2_CH_2_OCO2Li has an identical FTIR spectrum, which means that the data from experimental FTIR spectroscopy, for example in Ref. [41], also suggests the existence of CH_2_CH_2_OCO_2_Li in SEI film. As such, we believe that CH_2_CH_2_OCO_2_Li and (CH_2_OCO_2_Li)_2_ may co-exist in SEI film, in which CH_2_CH_2_OCO_2_Li is the inner layer. There are some experimental observations that the SEI films will cleave from the graphite surface once the battery is overcharged. We think this is because the overcharge impairs the adsorption binding between the CH_2_CH_2_OCO_2_Li and the graphite surface. As such, these observations also support our model. Recent in situ neutron reflectometry measurements have illustrated that the SEI layer has a LiOCO_2_C_2_H_5_ layer with a thickness of about 0.5 nm after 20 cyclic voltammetry cycles, later growing to about 1.0 nm on the copper surface. Considering the lengths of the molecules of CH_2_CH_2_OCO_2_Li and (CH_2_OCO_2_Li)_2_, which are 0.6 nm and 1.0 nm, respectively, this neutron reflectometry experiment evidences our theoretical findings.

One interesting thing that needs to be mentioned here is that the parallel orientation is more energetically favorable when the plane of C1-O1-O2 is parallel to the graphite layers. Our proposed network is formed through the “zipping” of carbonate branches through lithium ions, which is similar to the 3D network structure of the SEI proposed by Shkrob et al. from a spectroscopic study [74]. Such geometry makes the adsorption of (CH_2_OCO_2_Li)_2_ an extension or “growth” of the graphite layers, which allows Li intercalation into the graphite but blocks other molecular migration.

## 5. Conclusions

We have investigated the initial decomposition of EC and the formation of the innermost SEI layers on the surfaces of carbon anodes of Li-ion batteries using first-principles calculations. We have studied the adsorptions of EC, (CH_2_OCO_2_Li)_2_, and CH_2_CH_2_OCO_2_Li on the basal, zigzag, and armchair surfaces of graphite. We found that the homolytic ring opening of EC is the first step toward its decomposition. We examined the interaction between the initial product, CH_2_CH_2_OCO_2_^−^ and Li cations, and the graphite surface. We found a stable structure of CH_2_CH_2_OCO_2_Li, where all carbon atoms, oxygen atoms, and the lithium atom are in one plane, with a binding energy of 1.35 eV. This structure can be well adsorbed onto the graphite basal surface with an adsorption energy of −0.24 eV, with the Li atom 2.11 Å above the center of hexagon carbon atoms on the graphene.

The adsorption of CH_2_CH_2_OCO_2_Li will decrease with the concentration. CH_2_CH_2_OCO_2_Li has a strong adsorption on the graphite zigzag edge surface with an adsorption energy of −0.91 eV, due to the dangling carbon bonds, and suggests that the SEI film is primarily formed on the edge surface with CH_2_CH_2_OCO_2_Li as the innermost layer. During the study, we noticed that CH_2_CH_2_OCO_2_Li has the same character bonds, C-H, C-O, and C=C, as those of (CH_2_OCO_2_Li)_2_. These two molecules should have an identical FTIR spectra. As a result, it is hard to distinguish them by FTIR spectra, which is a main experimental method in studying the components of the SEI. We studied the adsorption of (CH_2_OCO_2_Li)_2_ and CH_2_CH_2_OCO_2_Li on the graphite surface using first-principles calculations. We propose an SEI-forming mechanism with the adsorption and bonding molecule CH_2_CH_2_OCO_2_Li with carbon atoms. Our proposed network is formed through a “zipping” of carbonate branches through lithium ions, agreeing with the 3D network structure of the SEI proposed by a spectroscopic study [74]. Such an SEI-forming mechanism on the graphite surface builds up the innermost layer of the SEI in EC-based electrolytes in a lithium-ion battery.

## Figures and Tables

**Table 1:** The results of the adsorption energies of CH_2_CH_2_OCO_2_Li and (CH_2_OCO_2_Li)_2_ on graphite basal, zigzag edge, armchair edge surfaces (units in eV).

	Basal Surface	Zigzag Edge Surface	Armchair Edge Surface
CH_2_CH_2_OCO_2_Li	−0.24	−0.91	−0.54
(CH_2_OCO_2_Li)_2_	−0.17	−0.49	−0.32

## Data Availability

The datasets generated during and/or analyzed during the current study are available from the corresponding author on reasonable request.

## Supplementary Materials

The following supporting information can be downloaded at: https://www.mdpi.com/article/10.3390/nano12203654/s1, Table S1: Size effect and *k*-mesh convergence for adsorption energy of EC on graphite surface; Figure S1: Various atomistic configurations of CH_2_CH_2_OCO_2_Li; Figure S2: Adsorption of EC on basal surface of graphite; Calculation of the adsorption energy and reaction energy.
