# Supplementary material for: First-Principles Insights on the Formation Mechanism of Innermost Layers of Solid Electrolyte Interphases on Carbon Anodes for Lithium-Ion Batteries
Source: Nanomaterials (Basel). 2022 Oct 18;12(20):3654. doi: 10.3390/nano12203654 (PMC9607018; doi:10.3390/nano12203654)
Supplement: Supplementary file 1 [file nanomaterials-12-03654-s001.zip › nanomaterials-1899978-supplementary.pdf]

Supplementary Materials

First-Principles Insights on the Formation Mechanism of Innermost Layers of Solid Electrolyte Interphases on Carbon Anodes for Lithium-Ion Batteries

Qing Peng [1,2,3]

[1] Physics Department, King Fahd University of Petroleum & Minerals, Dhahran 31261, Saudi Arabia; qing.peng@kfupm.edu.sa
[2] K.A.CARE Energy Research & Innovation Center at Dhahran, Dhahran 31261, Saudi Arabia
[3] Interdisciplinary Research Center for Hydrogen and Energy Storage,
King Fahd University of Petroleum and Minerals, Dhahran 31261, Saudi Arabia

Contents

1. **Table S1: Size effect and k-mesh convergence for adsorption energy of EC on graphite surface**

2. **Figure S1: various atomistic configurations of $CH_2CH_2OCO_2Li$.**

3. **Figure S2: Adsorption of EC on basal surface of graphite.**

4. **Calculation of the adsorption energy and reaction energy.**

Table S1: Size effect and k-mesh convergence for adsorption energy of EC on graphite surface. The adsorption energy is in unit of eV.

**Size**	***K*-mesh(3x3x1)**	***K*-mesh (6x6x1)**
4x4x1 (EC/C32)	-0.235	-0.231
5x5x1 (EC/C50)	-0.234	-0.233
6x6x1 (EC/C72)	-0.241	-0.241
7x7x1 (EC/C98)	-0.242	-0.242
8x8x1(EC/C128)	-0.246	-0.246
9x9x1(EC/C162)	-0.243	-0.246
10x10x1(EC/C200)	-0.242	-0.246

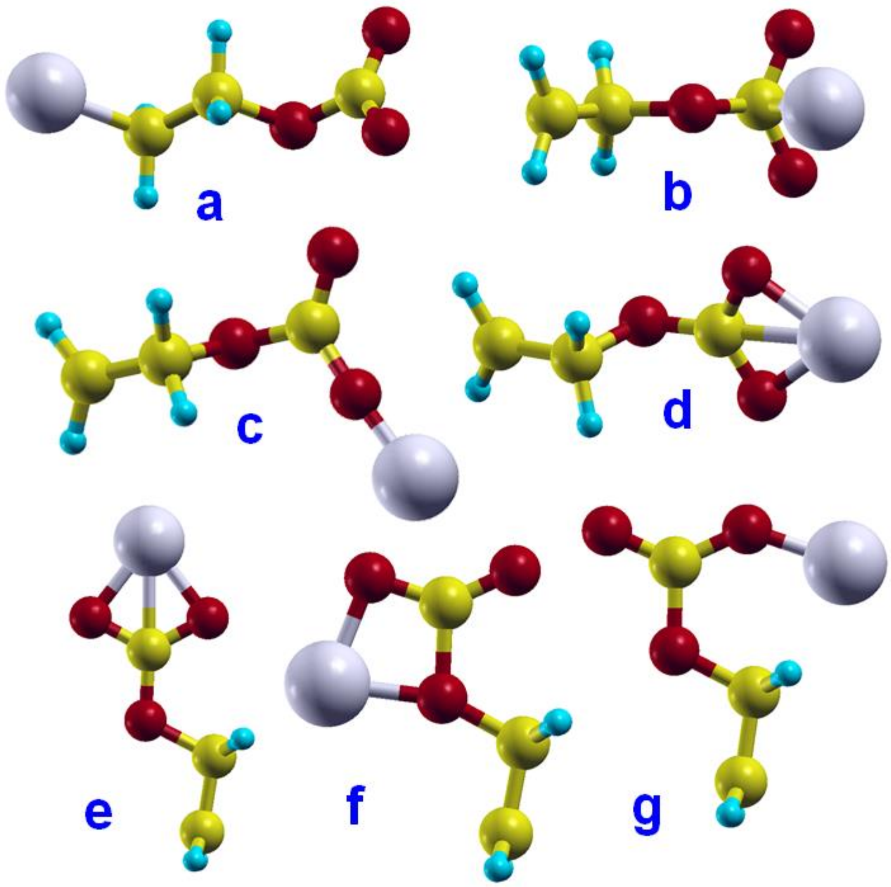

Figure S1: various atomistic configurations of $CH_2CH_2OCO_2Li$. The formation energy of the seven configurations are (a) 0.936 eV; (b) 0.197 eV; (c) -0.304 eV; (d) -0.939 eV; (e) -1.347 eV; (f) -1.218 eV, (g) -0.534 eV. Configure e has the lowest formation energy among the seven configurations.

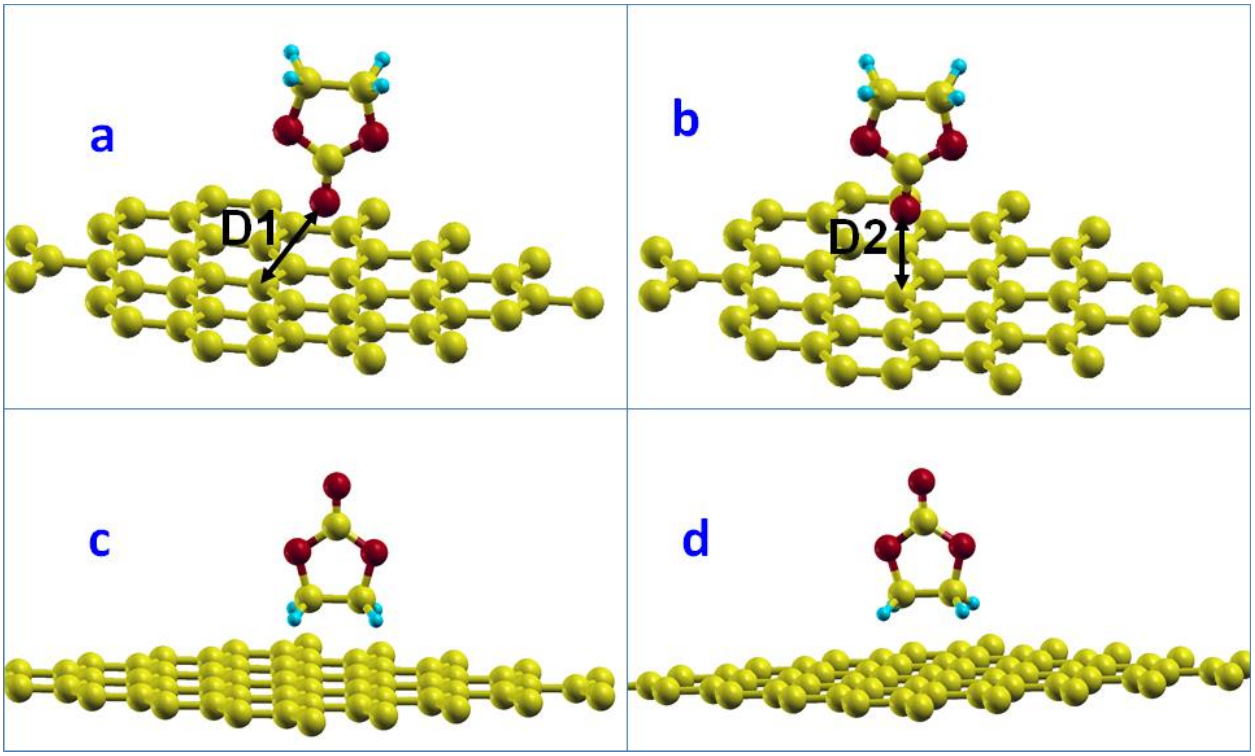

Figure S2: Adsorption of EC on basal surface of graphite. Four configurations were examined corresponding to the two orientations (Head and Tail) of EC molecules on two sites (Hollow and Top). (a) Head-Hollow site (b) Head-Top site (c) Tail-Hollow site (d) Tail-Top site. Configuration (a) has the largest adsorption energy amount the four configurations.

Calculation of the adsorption energy and reaction energy.

We are considering the following five chemical equations to demonstrate the calculations of the adsorption energy and reaction energy.

(1) Li + EC -> $CH_2CH_2CO_3Li$, ΔE = −1.35 eV

(2) 2Li + 2EC -> $(CH_2CO_3Li)_2$ + C_2H_4 , ΔE = −7.69 eV

(3) $2(CH_2CH_2CO_3Li)$ -> $(CH_2CO_3Li)_2$ + C_2H_4, ΔE = −4.99 eV

(4) $2(CH_2CH_2OCO_2Li)^*$ -> $(CH_2OCO_2Li)_2^*$ + C_2H_4, ΔE = −3.66 eV

The "*" denotes the case for adsorption on graphite surface.

The details of the deduction for these energies are follows.

We calculated the reaction energy as the following formula:

$$\Delta E = E(produce) - E(reactant)$$

Therefore, for Equation 1:

(1) Li + EC -> $CH_2CH_2CO_3Li$, ΔE = −1.35 eV
$\Delta E1$ =E($CH_2CH_2CO_3Li$)-E(Li)-E(EC)=-1.35eV

→E($CH_2CH_2CO_3Li$)= E(Li)+E(EC)-1.35 eV

(2) 2Li + 2EC -> $(CH_2CO_3Li)_2$ + C_2H_4 , ΔE = −7.69 eV
$\Delta E2$ =2E(CH_2CO_3Li)+E(C_2H_4)-2E(Li)-2E(EC)=-7.69eV

→2E(CH_2CO_3Li)= 2E(Li)+2E(EC)-E(C2H4)-7.69 eV

Therefore, for Equation (3): $2(CH_2CH_2CO_3Li)$ -> $(CH_2CO_3Li)_2$ + C_2H_4
The reaction energy is $\Delta E3$ =2E(CH_2CO_3Li)+E(C_2H_4)-2E($CH_2CH_2CO_3Li$)

=[2E(Li)+2E(EC)-E(C_2H_4)-7.69 eV] + E(C_2H_4) -2[E(Li)+E(EC)-1.35 eV]
= -7.69+2.70 eV
= -4.99 eV

Equation (3) also gives: E(C_2H_4) = 2E($CH_2CH_2CO_3Li$)- 2E(CH_2CO_3Li)-4.99 eV

For Equation 4, that is the adsorption on the graphite, $2(CH_2CH_2OCO_2Li)^*$ -> $(CH_2OCO_2Li)_2^*$ + C_2H_4,

The adsorption energy is calculated as follows:

$E_{ads} = E((CH_2OCO_2Li)_2 + graphite) - E((CH_2OCO_2Li)_2) - E(Graphite)$.

$E^1_{ads} = E((CH_2CH_2OCO_2Li)_2 + graphite) - E((CH_2CH_2OCO_2Li)_2) - E(Graphite)$ =2*(-0.91)=-1.82 eV

$E^2_{ads} = E((CH_2OCO_2Li)_2 + graphite) - E((CH_2OCO_2Li)_2) - E(Graphite)$=-0.49 eV

Then,
$\Delta E\_ads = E^2_{ads} - E^1_{ads}$ =2E(CH_2OCO_2Li)*-2E($CH_2CH_2OCO_2Li$)*- 2E(CH_2CO_3Li)+ 2E($CH_2CH_2CO_3Li$) =1.33 eV

→ [2E(CH_2OCO_2Li)*-2E($CH_2CH_2OCO_2Li$)*] =1.33 eV - 2E($CH_2CH_2CO_3Li$)+ 2E(CH_2CO_3Li)

Therefore, we can obtain the reaction energy of Equation (4) as:

ΔE4 =2E(CH_2OCO_2Li)*+E(C_2H_4)-2E($CH_2CH_2OCO_2Li$)*
=[1.33 eV - 2E($CH_2CH_2CO_3Li$)+ 2E(CH_2CO_3Li)] + [2E($CH_2CH_2CO_3Li$)- 2E(CH_2CO_3Li)-4.99 eV] = -3.66 eV
